# Enhanced interfragmentary stability and improved clinical prognosis with use of the off-axis screw technique to treat vertical femoral neck fractures in nongeriatric patients

**DOI:** 10.1186/s13018-021-02619-8

**Published:** 2021-07-31

**Authors:** Dajun Jiang, Shi Zhan, Qianying Cai, Hai Hu, Weitao Jia

**Affiliations:** Department of Orthopedic Surgery and Orthopedic Biomechanical Laboratory, Shanghai Jiao Tong University Affiliated Sixth People’s Hospital, Shanghai, 200233 People’s Republic of China

**Keywords:** Vertical femoral neck fractures, Internal fixation, Clinical study, Biomechanical study, Cross-screw technique

## Abstract

**Background:**

The optimal internal fixation strategy for vertical femoral neck fractures (VFNFs) in nongeriatric patients remains uncertain. Therefore, the purpose of this study was to compare the clinical prognoses and underlying mechanical characteristics of a novel off-axis screw technique with dynamic hip screws (DHSs) and three traditional parallel screws.

**Methods:**

This study included a clinical investigation and a patient-specific finite element analysis (FEA). In the clinical investigation, VFNF patients were grouped by fixation type: (1) use of three parallel screws (G-TRI); (2) augmentation with an off-axis screw (G-ALP); and (3) DHS with an anti-rotational screw (G-DHS). Fixation failures (nonunion, femoral neck shortening (FNS), varus deformation, screw cut-out) and avascular necrosis (AVN) consequent to the three types of fixations were compared. In the FEA, twenty-four fixation models with the three fixation types were created based on the data of eight healthy volunteers. Models were assessed under walking conditions. Stiffness, interfragmentary motion (IFM), and implant stress were evaluated.

**Results:**

In the clinical investigation, the fixation failure rate was significantly (*p* < 0.05) lower in G-ALP (18.5%) than in G-DHS (37.5%) and G-TRI (39.3%). No significant difference in AVN was observed among the three fixation groups. In the FEA, stiffness and implant stress in the G-DHS models were significantly (*p* < 0.05) higher, and the IFM of G-ALP was significantly (*p* < 0.05) lower among the groups.

**Conclusions:**

Among fixation types for VFNFs, the off-axis screw technique exhibited better interfragmentary stability (lowest IFM) and a lower fixation failure rate (especially FNS). Analyzing interfragmentary stability in biomechanical experiments is more consistent with clinical prognosis than construct stability for VFNFs, suggesting that internal fixations should aim for this outcome.

**Supplementary Information:**

The online version contains supplementary material available at 10.1186/s13018-021-02619-8.

## Background

The treatment of vertical femoral neck fractures (VFNFs) in patients younger than 60 years of age is problematic [1]. This is primarily because of the high-energy violent nature of the trauma, an inherently unfavorable blood supply to the femoral neck, inherent biomechanical instability, and use of an inappropriate fixation strategy. Typical prognoses for internal fixations are disappointingly poor, with fixation failure rates reaching as high as 41.9% and avascular necrosis rates reaching as high as 16–21% [2, 3]. These complications severely impair functional outcomes and ultimately result in arthroplasties, which lead to a lower quality of life for younger patients. An optimal fixation strategy for VFNFs is required to prevent these clinical complications, but the topic has been debated for years.

According to a 2014 questionnaire study of 573 orthopedic surgeons [1], dynamic hip screws (DHSs) and cannulated screws are two of the most widely used devices for treating VFNFs. Recent studies [4, 5] have recommended DHSs over cannulated screws if the femoral neck fracture is vertically oriented, due to its fixed-angled nature and greater stiffness [6]. Biomechanically, a DHS can be described as a “load-bearing” device, which means that the implant can withstand more load from the deforming force and thus is more suitable for unstable fractures [4]. In contrast, cannulated screws can be described as a “load-sharing” device, which is better for stable fractures.

However, the definitive recommendation that DHSs are better than cannulated screws in VFNFs is still to be presented because clinical evidence is controversial [7–10]. A recent meta-analysis [10] revealed that cannulated screws have similar mortality, revision, and nonunion rates as DHSs and are superior with respect to avascular necrosis (AVN). In addition, recent studies [11] have shown that augmentation with an off-axis screw for parallel screw fixation significantly increases the resistance to shear deformation force [11] and reduces the complication rate in clinical follow-up [12]. Consequently, the question arises: Is it possible that fixation with a load-sharing device can also achieve a satisfactory clinical outcome for VFNFs by modifying the standard screw configuration?

The purpose of this study was to compare the clinical prognoses and underlying mechanical characteristics of the novel off-axis screw technique with DHS and three traditional parallel screws in treating vertical femoral neck fractures. We hypothesized that in comparison with the other fixation strategies, the novel off-axis axis technique can provide better interfragmentary stability and obtain a lower fixation failure rate.

## Methods

This study comprised two parts: a retrospective clinical investigation and a patient-specific finite element analysis (FEA).

### Part 1: Retrospective Clinical Investigation

**Fig. 1:**
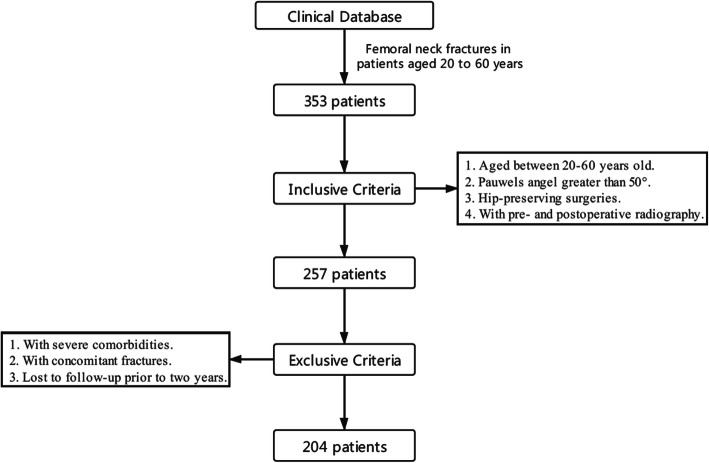
Patient selection process

The retrospective cohort study included patients with a diagnosis of VFNFs who were treated with internal fixations at one orthopedic ward of our institution between December 2013 and December 2017. The following patients were eligible: (1) aged between 20 and 60 years old; (2) had a Pauwels angle greater than 50° (i.e., vertical-oriented type, VFNF); (3) treated with hip-preserving surgeries; and (4) received pre- and postoperative radiography. The Pauwels angle was measured using the modified method based on preoperative antero-posterior X-rays [13]. The following patients were excluded: (1) those with severe comorbidities, including osteoarthritis, osteoporosis, cerebrovascular disease, and diabetes, among others; (2) those who additionally had femoral shaft, subtrochanteric, intertrochanteric, or contralateral fractures along with a VFNF; and (3) those with less than 2 years of follow-up. The strict patient selection process was beneficial for improving the representativeness of the participants and reducing selection bias. Finally, a total of 204 patients were included in the clinical investigation (Fig. 1), and all gave written informed consent to participate. This study was approved by the local institutional ethics review board (No. 2016-143) and was carried out in accordance with the World Medical Association Declaration of Helsinki [14].

Among the 204 patients with VFNFs, 107 patients were treated with three parallel 6.5 mm cannulated screws (Stryker) (denoted as group G-TRI); 65 patients were treated with three parallel screws augmented with an off-axis screw (denoted as group G-ALP); and 32 patients were treated with DHSs (Depuy Synthes) plus one anti-rotational screw (denoted as group G-DHS). To further prevent selection bias, the choice of internal fixation was determined by the treating surgeons; this choice was unrelated to the patients’ condition and fracture severity and was also in respect of the patients’ autonomy and wishes.

In all of the surgeries, closed reduction was attempted first, and then the reduction quality was evaluated based on intraoperative fluoroscopy imaged in the AP and lateral planes. Acceptable reductions (displacement < 5 mm, angulation < 10°) were defined using the Haidukewych criteria [15]. For fractures with unacceptable reduction, open reduction was performed using the modified Smith-Peterson approach. After an acceptable reduction was achieved, an incision was made laterally according to the type of internal fixation device used. In G-TRI, three 6.5 mm partial thread screws were implanted in parallel in a regular or inverted angular configuration. In G-ALP, the off-axis screw was inserted ahead of the parallel screws from 2 mm proximal to the femoral vastus ridge, directing it toward the inferior calcar. In G-DHS, a 6.5 mm partial thread screw was inserted before implanting the lag screws and the fixed-angled barrel plate.

Postoperatively, patients were discouraged from engaging in any weight-bearing activities during the first 3 months of recovery. Thereafter, they were allowed to gradually engage in partial weight-bearing activities only if their radiographs revealed an acceptable bone union. Patients were scheduled for postoperative follow-up 6 weeks, 3 months, and 1 year after fixation surgery. Thereafter, they were evaluated once per year. At each point of follow-up, AP and lateral radiography was performed for assessment.

The primary outcome was fixation failure, including nonunion (NU), femoral neck shortening (FNS), varus deformation (VD), and cut-out [2]. NU was confirmed when bone healing was not achieved within 6 months. FNS was defined as > 10 mm shortening of the femoral neck length, while VD was defined as a > 10° decrement in the femoral neck-shaft angle [16]. All variables included in the primary outcome were objective parameters observed in radiographs, thus reducing possible recall bias.

The secondary outcome was AVN and functional recovery. AVN was evaluated radiographically by using the Ficat method [17]. Functional recovery was evaluated using the Harris Hip Score (HHS) at 2 years after the operation. Any patient with a secondary procedure within 2 years was included only up to the follow-up for the primary procedure.

To reduce confounding bias, variables including the patients’ demographic characteristics (age, sex), fracture severity (initial displacement, Pauwels angle), and surgical information (reduction method, reduction quality, intraoperative blood loss, operation duration) were also recorded as baseline information. All variables were assessed by two independent surgeons (DJ, Jiang and H, Hu) and were further determined by another senior surgeon (WT, Jia).

### Part 2: Patient-Specific FEA

**Fig. 2:**
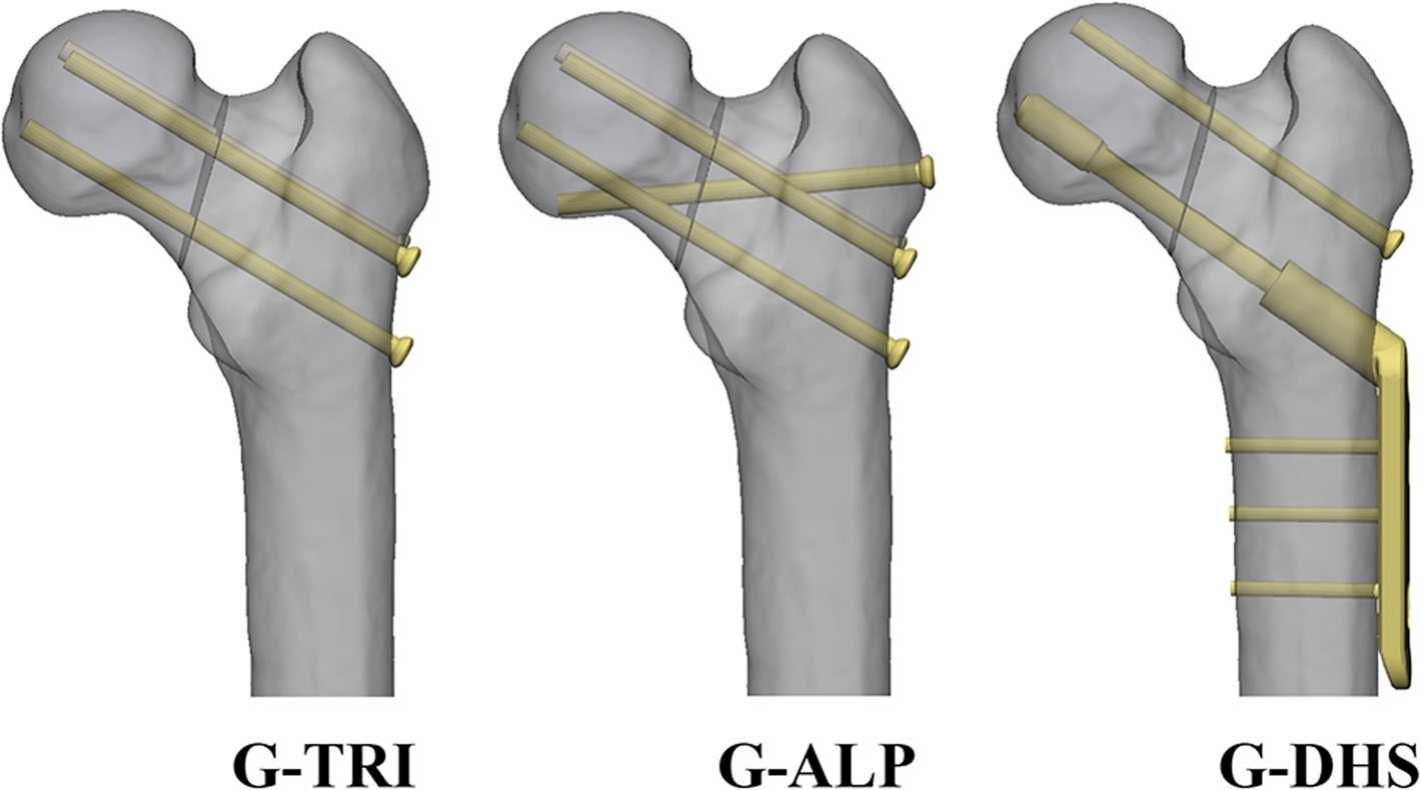
The three groups of fixation models studied in the clinical and biomechanical investigations included the Alpha (G-ALP), Inverted Triangle (G-ITR), and Triangle (G-TRI) groups. Three parallel screws were all positioned dispersedly, at 2.5 mm to the cortex and 5 mm distal to the subchondral bone in the femoral head. The off-axis screw in G-ALP was implanted 5 mm proximal to the most prominent part of the great trochanter and targeted at the inferior femoral head-neck junction. DHS was implanted inferiorly. The anti-rotational screw was 1.5 cm parallel and superior to the nail

We also conducted a patient-specific FEA based on data from 8 healthy volunteers, ranging in age from 20 to 60 years old (Additional file 1). All volunteers were requested to undergo a full-length 0.625mm-thick CT scan of the lower extremities after daily calibration. The patient-specific FEA models were developed based on CT images in Mimics software (Version 19.0, Materialise, Leuven, Belgium) and further digitally osteotomized with a Pauwels angle of 70° in 3-Matic software (Version 11.0, Materialise, Leuven, Belgium). The three internal fixation strategies we tested in these models were (1) triangle fixation (G-TRI); (2) triangle screws with an off-axis screw (G-ALP); and (3) DHS (G-DHS). These were compared in each patient-specific fracture model (Fig. 2). The digital construction of these devices was created in SolidWorks 2017 (Dassault Systèmes SolidWorks Corporation, Waltham, MA, USA) and assembled in 3-Matic software. To control for confounding variables of surgical quality, all fixation devices were digitally implanted based on the same standard criteria (Fig. 2) [11, 18].

All assemblies were meshed into 1-mm equal sized facets and converted into 4-node linear tetrahedron (C3D4) solid elements in Hypermesh 13.0 (Altair Engineering, Troy, MI, USA). The 1 mm mesh size was chosen according to a previous convergence test [19] of the proximal femur. Each solid model, containing approximately 520,000 elements (1-mm-sized) and 110,000 nodes, was then exported into Abaqus 6.13 software (Simulia Corp, USA) as inp. format files. The properties of all bone and implant models were assumed to be linear elastic materials. The density of cortical and cancellous bone was determined by calculating their Hu values based on computed tomography (CT) scans using the formula described previousl y[20].

$$ \uprho \left(\mathrm{g}/{\mathrm{cm}}^3\right)=0.000968\ast \mathrm{HU}+0.5 $$

$$ \mathrm{If}\ \rho <1.2\ \mathrm{g}/{\mathrm{cm}}^3,E=2014\ast \rho \hat{\mkern6mu} 2.5\ \left(\mathrm{MPa}\right),\nu =0.2. $$

$$ \mathrm{If}\ \rho >1.2\ \mathrm{g}/{\mathrm{cm}}^3,E=1763\ast \rho \hat{\mkern6mu} 3.2\ \left(\mathrm{MPa}\right),\nu =0.32. $$

**Fig. 3:**
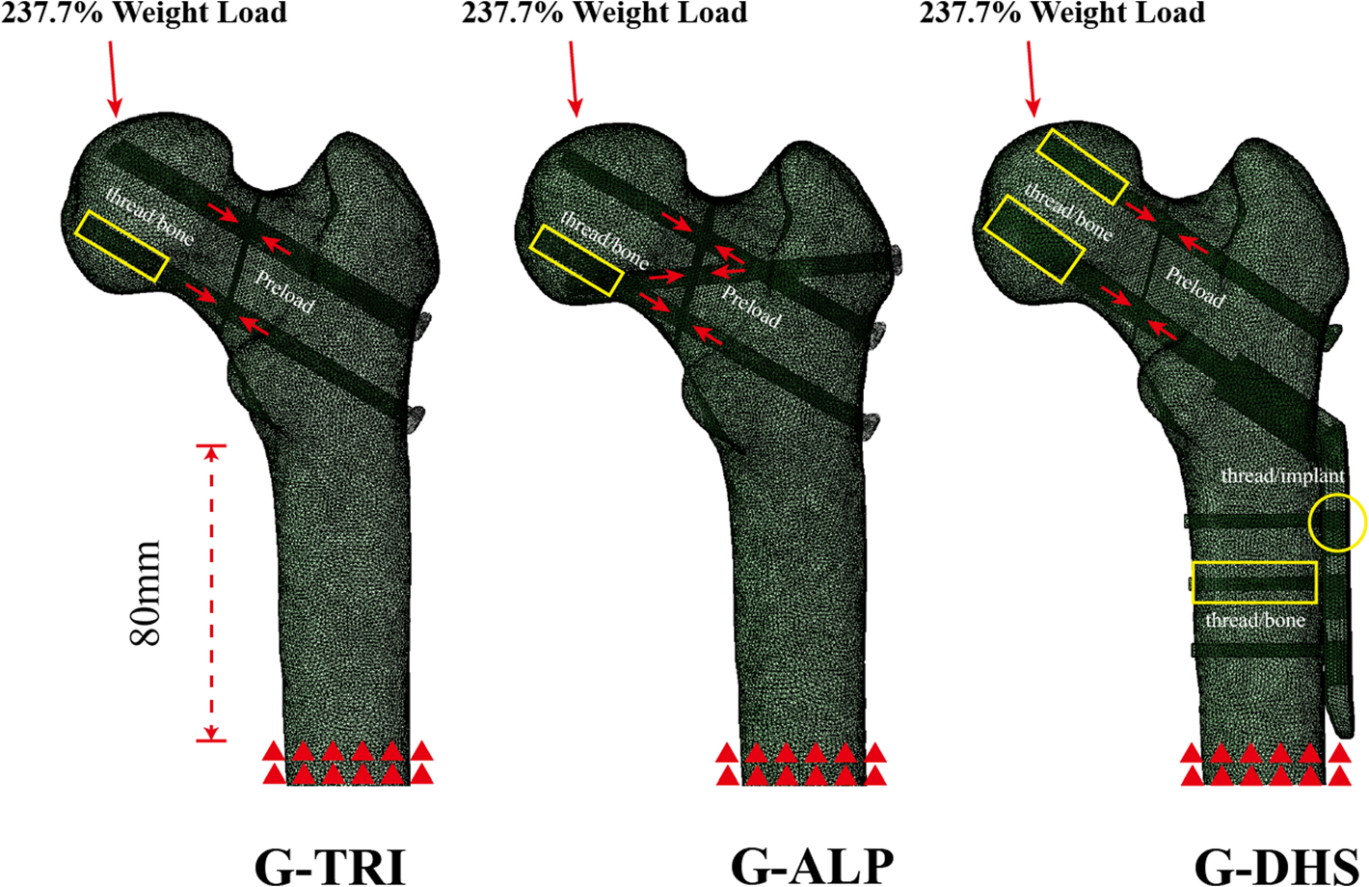
The schematic diagram of finite element analysis. During the finite element analysis, thread-bone (yellow boxes) and thread-implant (yellow circle) interfaces were tied, while others were set to slide contact. All fixation models were constrained to within 80 mm distal from the lesser trochanter and subjected to 237.7% body weight loading along the femoral mechanical axis. A preload of 224 N for cannulated screws and 591 N for DHSs was applied ahead of the weight load

For cannulated screws, we used values for screws made of titanium (Ti-6L-4V), which has a Young’s modulus (*E*) of 110,000 MPa and a Poisson’s ratio of 0.3 [21, 22]. For the DHS device, we used values for stainless steel, which has a Young’s modulus (*E*) of 193,000 MPa and Poisson’s ratio of 0.31 [23]. As shown in Fig. 3, to simulate the mechanical nature of these implants, thread-bone/implant interfaces were tied while others were set to slide contact. Tie contacts were assigned to thread-bone interfaces and screw-barrel plates. Sliding contacts were assigned to fracture interfaces with a frictional coefficient of 0.46 [24]. The other interfaces were set as self-contacts with a frictional coefficient of 0.3 [24]. All fixation models were constrained to within 80 mm distal from the lesser trochanter and subjected to 237.7% body weight loading [25], in line with the femoral mechanical axis. The node on the weight-bearing region of the femoral head that intersects the mechanical axis was set as the loading position. The dynamic compression effect of cannulated screws and lag screws was simulated by using a preload of 224 N for cannulated screws and 591 N for DHSs, which was the same value per mm^2^ that we described previously [11]. The above finite element simulation process was validated using cadaveric bone in our previous biomechanical test [11], showing a relative coefficient of 0.78–0.94 in terms of strain distribution.

Stiffness, IFM, and implant stress (von Mises stress) were analyzed as biomechanical parameters. Stiffness was calculated by dividing the loading by the displacement of the applying node. To overcome the drawback of the “center point fallacy” in previous measurements [26], the IFM of all nodes on both fracture surfaces was calculated, and then the mean IFM value of all nodes was the parameter that was compared among the three groups [11]. The IFM of each paired node was calculated based on the formula in a previous study [11].

### Statistical analysis

Both the clinical and biomechanical statistical analyses were performed with SPSS 24 (IBM Corp. Released 2016. IBM SPSS Statistics for Windows, Version 24.0. Armonk, NY: IBM Corp). Categorical variables are expressed as frequencies and were compared using the chi-squared test. Continuous variables are presented as means and standard deviations and were checked for normality using the Shapiro-Wilk test (biomechanical analysis), Kolmogorov-Smirnov test (clinical analysis), and Q-Q plot. Parametric continuous variables were compared using one-way ANOVAs (randomized block one-way ANOVAs for biomechanical analysis), while nonparametric continuous variables were compared using the Mann-Whitney test. Univariate analysis was performed first, and all variables related to the primary outcome were considered to be adjusted in a multivariate regression analysis. The interobserver reliability of clinical outcomes was tested using intraclass correlation coefficients (ICCs). Statistical significance was set at *p* < 0.05.

## Results

Overall, all data in this study obeyed a normal distribution (*p* > 0.05). All variables in the clinical investigations had an ICC value over 0.7, indicating that these results were convincing and reproducible [27].

In the clinical investigation, 204 VFNF cases with a mean age of 45.4 (± 10.4) years were finally included in the analysis. These patients underwent an average follow-up of 45.0 ± 20.7 months, and none of them had missing data (Fig. 1). Baseline information was statistically indistinguishable (*p* > 0.05) in terms of age, sex, initial displacement, Pauwels angle, reduction method, and reduction quality (Table 1). Intraoperative blood loss was significantly greater in G-DHS than in G-TRI and G-ALP (*p* < 0.001) and was similar between G-TRI and G-ALP. The operation duration was significantly different between the two groups and was longest for G-DHS (119.4 ± 41.9 min), intermediate for G-ALP (75.3 ± 36.5 min), and shortest for G-TRI (57.5 ± 26.5 min). Notably, there was no significant association between fixation failure and intraoperative blood loss (*p* = 0.545) or operation duration (*p* = 0.283).

**Table 1:** Baseline characteristics of the included patients with vertical femoral neck fractures

Variable	Internal fixation type*			***P*** value
	TRI	ALP	DHS	
**Participants**	107	65	32	
**Age**	44.5 ± 10.7	44.6 ± 10.6	48.2 ± 8.2	0.25
**Sex**				
**Female**	47	28	9	0.262
**Male**	60	37	23	
**Initial displacement**				
**Nondisplaced**	22	15	6	0.87
**Displaced**	85	50	26	
**Pauwels angle**	56.6 ± 7.4	58.0°± 7.0	59.7°± 10.7	0.226
**Reduction method**				
**Open**	26	15	8	0.65
**Closed**	81	50	24	
**Reduction quality**				
**Excellent-Good**	83	53	25	0.82
**Fair-Poor**	24	12	7	
**Intraoperative blood loss**	61.6 ± 52.9	86.3 ± 78.2	243.4 ± 194.8	< 0.001
**Operation duration**	57.5 ± 26.5	75.3 ± 36.5	119.4 ± 41.9	< 0.001

**Fig. 4:**
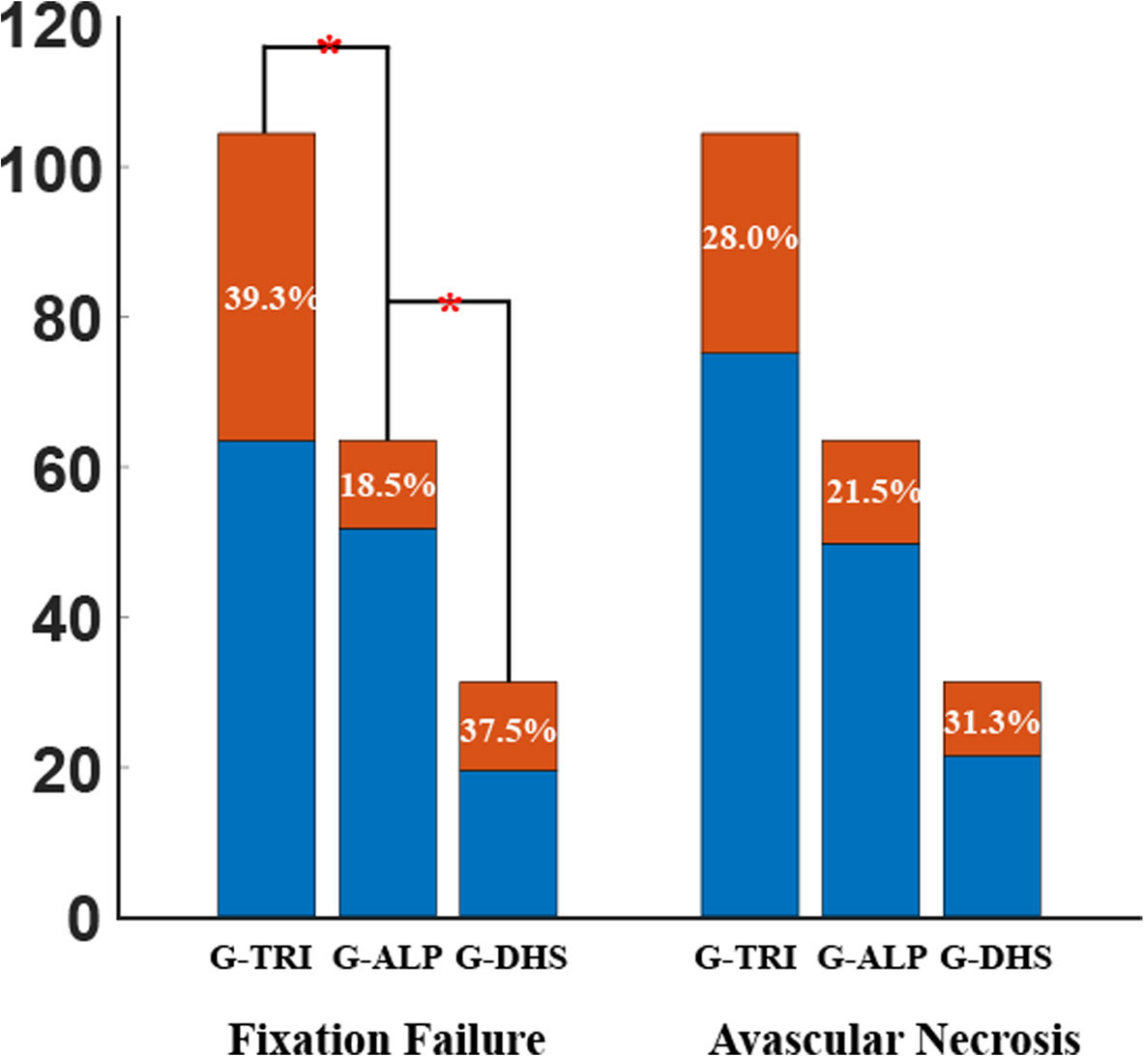
Fixation failures and avascular necrosis in the three groups. The fixation-failure rate was significantly lower in G-ALP than in G-TRI and G-DHS. The avascular necrosis rate was also the lowest numerically in G-ALP but was not statistically significant. The height of each bar represents the total number of patients in each group, while the rust-colored section in each bar represents the proportion of patients with complications within the group, and the blue-colored section represents the proportion without complications. Red asterisks indicate significant differences

**Fig. 5:**
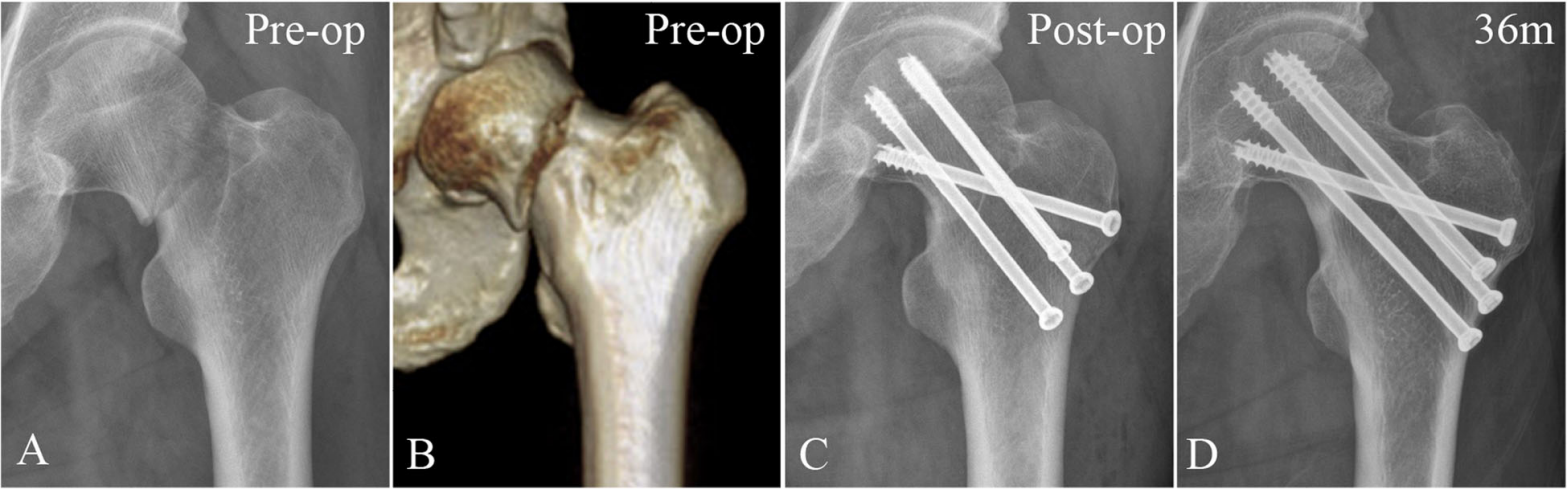
3-D reconstructed model of hip and AP radiographs of fracture and treatment fixation in a 49-year-old male with a vertical femoral neck fracture. **a**, **b** Preoperative radiograph and reconstructed model showing the vertical femoral neck fracture. **c** AP radiograph showing the initial treatment with three parallel screws augmented with an off-axis screw. **d** AP radiograph of the same patient taken 36 months postoperatively, revealing bone union without any complications

**Fig. 6:**
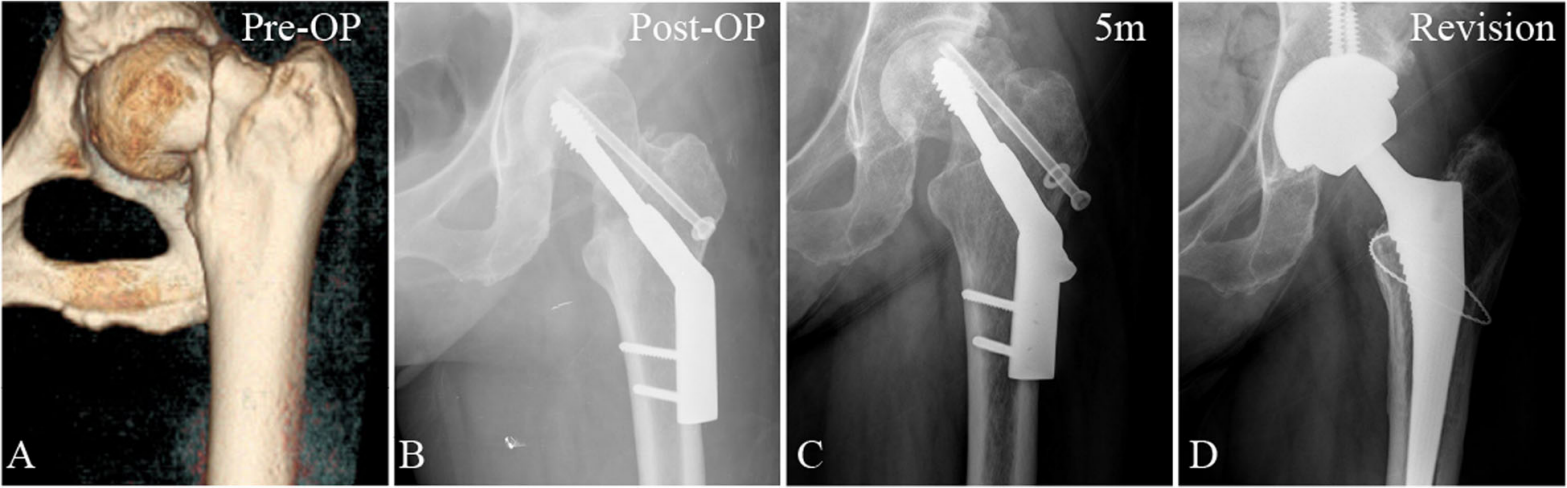
3-D reconstructed model of hip and AP radiographs of fracture, treatment fixation and revision implants in a 59-year-old woman with a vertical femoral neck fracture. **a** Reconstructed model (AP view) from preoperative CT images showing the location of the fracture. **b** AP radiograph showing initial treatment with a dynamic hip screw with an anti-rotational screw. **c** AP radiograph of the same patient taken 5 months after initial surgery, revealing severe screw withdrawal, femoral neck shortening, varus deformation, and delayed union. **d** AP radiograph of the same patient after revision operation with arthroplasty

**Fig. 7:**
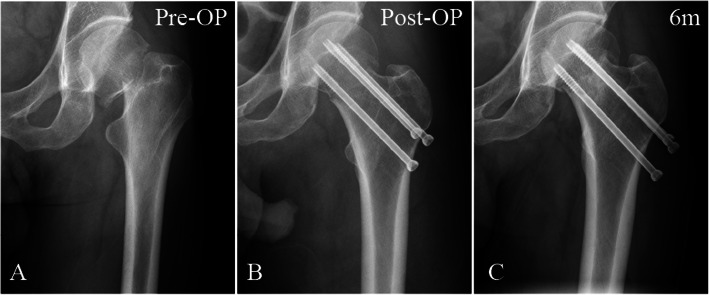
AP radiographs of a parallel screw fixation in a 58-year-old man. **a** Preoperative radiograph showing a vertical femoral neck fracture. **b** Postoperative radiograph showing treatment with three cannulated screws. **c** Radiograph taken 6 months postoperatively in the same patient, showing severe screw withdrawal, femoral neck shortening, and delayed union, as seen in the fracture line. *Pre*-*OP* preoperative, *Post*-*OP* postoperative, *6 m* 6 months after surgery

Table 2 shows the distribution of clinical prognoses across the three groups. As shown in Fig. 4, fixation failures were significantly (*p* = 0.015) lower in G-ALP (18.5%) (Fig. 5) than in G-DHS (37.5%) (Fig. 6) and G-TRI (39.3%) (Fig. 7). The rate of AVN was similar (*p* > 0.05) among G-TRI (28.0%), G-DHS (31.3%), and G-ALP (21.5%). The HHS was significantly higher in the G-ALP group than in the G-ITR group (*p* = 0.037) but showed nonsignificant differences between the other groups (*p* > 0.05).

**Table 2:** Distribution of clinical prognoses of the three fixation groups after at least 2 years of follow-up

Outcome, n (%)	G-TRI (***n*** = 107)	G-ALP (***n*** = 65)	G-DHS (***n*** = 32)	***P*** value*
**Fixation failure**	42 (39.3%)	12 (18.5%)	12 (37.5%)	0.015*
**Nonunion**	13 (12.1%)	2 (3.1%)	4 (12.5%)	0.111
**Femoral neck shortening**	34 (31.8%)	8 (12.3%)	9 (28.1%)	0.015*
**Varus deformation**	22 (20.6%)	7 (10.8%)	3 (9.4%)	0.13
**Cut-out**^**a**^	10 (9.3%)	3 (4.6%)	3 (9.4%)	0.503
**Avascular necrosis**	30 (28.0%)	14 (21.5%)	10 (31.3%)	0.516
**Harris Hip Scores**	80.2±14.7	87.8±10.4	82.2±14.3	0.103^b^

*p < 0.05

^a^Cut-out is defined as protrusion of the screw out of the femoral head

^b^HHS was significantly different between G-ALP and G-ITR (*p* = 0.037)

**Fig. 8:**
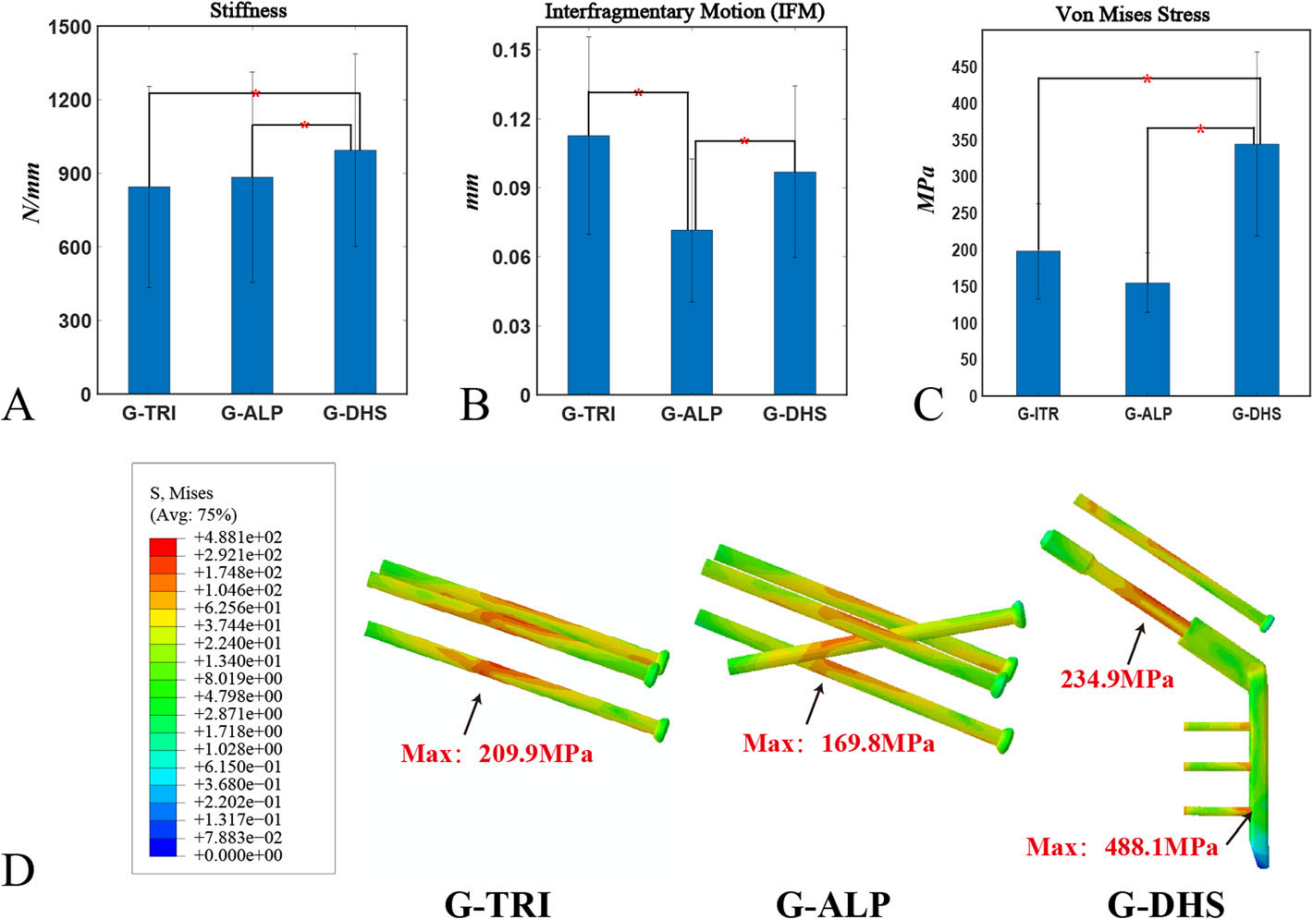
Results of patient-specific finite element analysis (FEA) in the biomechanical part of the study. **a**–**c** Comparison of mean stiffness (N/mm), interfragmentary motion (mm), and implant stress (MPa) for the three types of fixations. Error bars indicate the SD. **d** Von Mises distribution of the three fixation strategies

In the biomechanical analysis (Fig. 8A), the stiffness of G-DHS fixations (993.4 ± 392.3 N/mm) was significantly higher than that of G-ALP (883.6 ± 427.8 N/mm, *p* = 0.004) and G-TRI (844.6 ± 408.5 N/mm, *p* < 0.001) fixations. No significant (*p* = 0.268) difference was observed between the G-ALP and G-TRI models in terms of stiffness. IFM was significantly lower in G-ALP (0.071 ± 0.031 mm) than in G-DHS (0.097 + 0.037 mm, *p* =0.037) and G-TRI (0.113 ± 0.043 mm, *p* = 0.02) (Fig. 8B). G-DHS fixation had significantly greater implant stress (343.6 ± 125.9 MPa) than the G-TRI (196.8 + 64.8 MPa, *p* = 0.003) and G-ALP (154.0 + 40.5 MPa, *p* < 0.001) fixations (Fig. 8C, D).

## Discussion

The present study performed a comprehensive clinical and biomechanical evaluation of the most commonly used internal-fixation device s[1], comparing their relative rates of complications after a 2-year follow-up. We found that compared to the “load-bearing” device DHS, the “load-sharing” type of device, namely, the cannulated screw, exhibited a significantly lower fixation failure rate (18.5% vs. 37.5%) when it was implanted using the off-axis technique. By investigating their biomechanical characteristics, we found that DHS fixation had the best construct stability, while the off-axis screw technique had the best interfragmentary stability. The analysis of the corresponding clinical prognosis showed that interfragmentary stability (IFM) may better reflect fixation-failure outcomes, especially severe FNS.

Generally, cannulated screws are the most commonly used devices to treat femoral neck fractures [28, 29]. This preference is related to the advantages of accomplishing fixation with minimally invasive surgery, good dynamic interfragmentary compression, ease of screw implantation, and low expenses. However, cannulated screws lack a fixed-angle construction, thus exhibiting relatively lower stiffness [4, 6].. Therefore, they are described as “load-sharing” implants. In VFNFs, the biomechanical environment is not conducive to bone healing due to high shear, compressive, tensile, and torsional strain across the fracture site. In the present study, we found that three parallel screws were unsatisfactory for repairing these fractures, leading to a fixation-failure rate as high as 36.1% and an AVN rate of 25.8% in our series. These two complication rates are comparable with those reported in other clinical investigations [2, 13].

In contrast to cannulated screws, DHSs are load-bearing implants with the advantages of rigid fixed-angle mechanical construction. The present study demonstrated that the stiffness of DHSs is significantly greater than that of cannulated screws, with or without an off-axis screw. This, and the finding that patients in the G-DHS group had significantly higher implant stress than the other groups, provided further evidence of the load-bearing character of DHSs. Current recommendations for using DHSs rather than cannulated screws are based more on mechanical considerations than clinical evidence [7, 8], indicating that an evidence gap may exist between current biomechanical and clinical studies of VFNFs and how they relate. In our series, the use of DHS or TRI fixations was not significantly different in terms of fixation failure, which is in accordance with the findings of the FAITH study [9]. In addition, the occurrence of AVN was similar among all fixation groups in the present analysis; however, a previous FAITH study [9] reported a higher AVN rate in patients who underwent DHS fixation. This is likely due to the greater intraoperative blood loss and operation duration in the DHS procedure, which was detected in the current analysis. Therefore, the use of load-bearing devices, such as DHSs, in treating VFNFs is still unsatisfactory. It is necessary to develop novel devices or to modify traditional fixation strategies to improve the clinical prognosis of VFNFs.

What is the benefit of an additional off-axis screw? We found that adding an off-axis screw to parallel screws improved stability while still being minimally invasive and inexpensive. Our study showed that despite achieving similar stiffness, the off-axis screw technique showed significantly lower IFM, a lower fixation-failure rate and improved functional scores than parallel screws alone. As demonstrated in our previous study [11], the main mechanical advantage of adding the off-axis screw is to temper the “sliding effect,” improving bone purchase and cortical support. The additional screw also provided increased resistance to shearing deformation forces and ultimately decreased IFM. Until now, no previous direct clinical and biomechanical comparisons have been performed between the off-axis screw and DHS techniques. Despite a significantly lower stiffness, the present clinical observation showed that the off-axis screw technique is similar to the load-bearing implant (DHS) in terms of nonunion, varus deformation, cut-out, and avascular necrosis. Furthermore, this technique can significantly reduce the occurrence of FNS and potentially improve functional scores. According to the present biomechanical analysis, a significantly lower IFM in off-axis screw fixation can prevent a high strain environment, which is detrimental for bone repair [30] and predisposes patients to fixation failure, such as FNS. Severe FNS is a critical factor of the lower functional scores [31, 32], which may be due to abductor moment reduction as well as irritation from protruding screws.

**Fig. 9:**
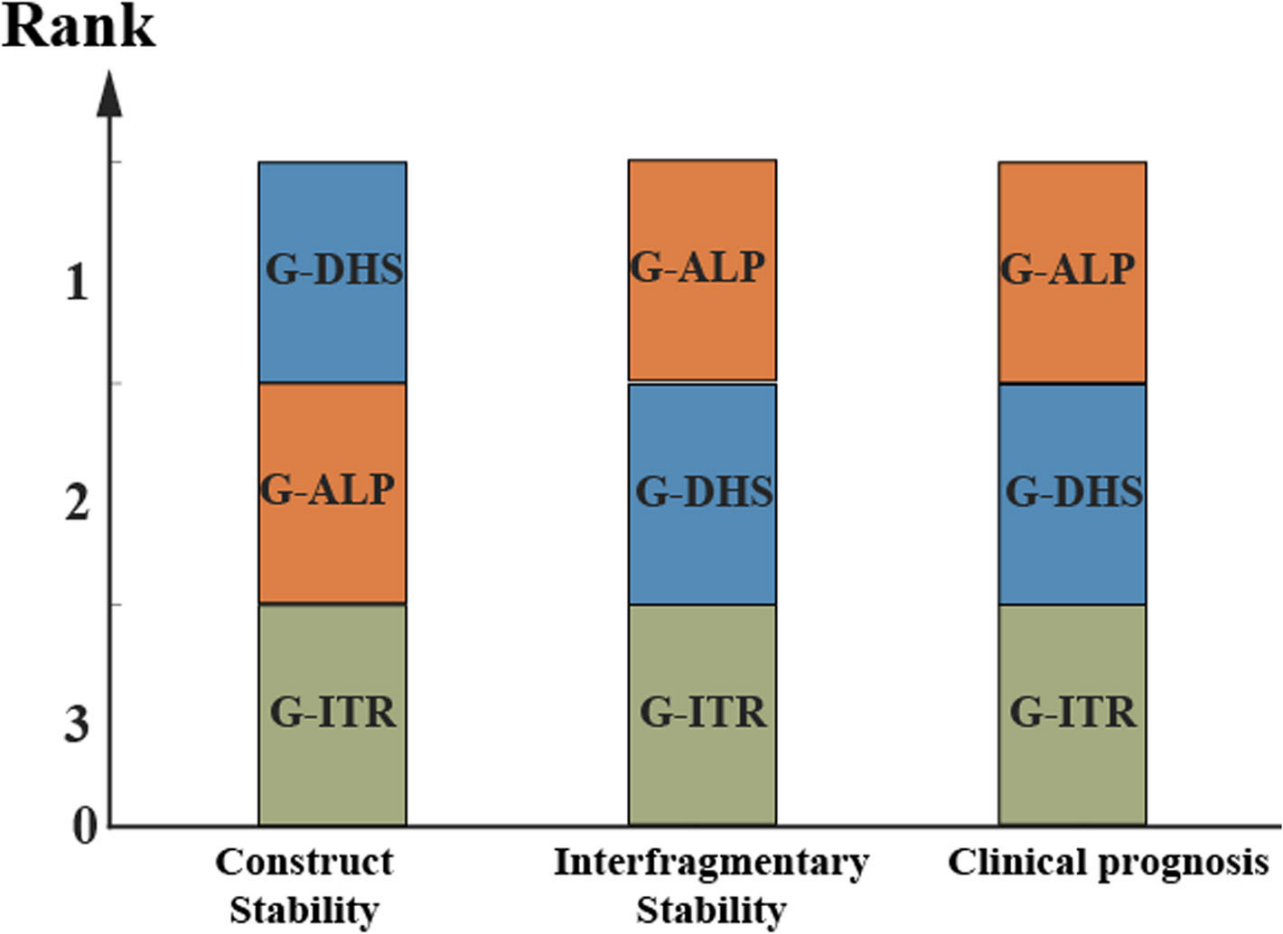
Rank order of the construct stability, interfragmentary stability, and clinical prognosis (fixation failure) of the three fixation types. Construct stability, interfragmentary stability, and clinical prognosis were evaluated separately by stiffness, IFM, and fixation failure

Generally, both stiffness and IFM are two important but separate parameters in static mechanical experiments. Stiffness, the most historical and commonly reported mechanical characteristic [26], is defined by the ratio between the deforming load and displacement along the loading direction [33] and represents “construct stability” but cannot isolate the fracture surface. In contrast, IFM, an increasingly common output parameter studied in recent years, is defined as the movement within the fracture gap, reflects the interfragmentary stability and can more directly reflect stability after fixation in real-world situations [26]. Few contemporary studies have investigated the intrinsic connection and differences between the two types of stability parameters and their relationship to clinical prognosis. Although DHS fixation is stiffer because of its fixed-angle characteristic, it is actually a “two-point” fixation across the fracture site and thus exhibits relatively poorer interfragmentary stability. In contrast, despite having less stiffness, the off-axis screw technique may be characterized as a “four-point” fixation and can constitute a crossed configuration; this results in satisfactory interfragmentary stability. According to the results of our clinical investigation, the fixation strategy that had greater interfragmentary stability, rather than construct stability, led to a better clinical prognosis (Fig. 9). Femoral neck fracture is an intra-articular fracture and requires absolute stable internal fixation and primary healing. It is necessary for internal fixations to eliminate any possible interfragmentary movement, a possibility that can be better evaluated by measuring IFM. Consequently, for fractures that require absolute stable fixation and primary healing, measuring IFM in biomechanical studies may be more informative.

## Limitations

We acknowledge that this study has some weaknesses. One limitation is this investigation was performed in one tertiary medical center, which may restrict its generalizability. Further clinical investigation recruiting multiple centers from a variety of countries is necessary to clarify this topic. Additionally, the cases were retrospectively collected in one medical center. Potential selection and confounding biases may still exist in the analysis. Future prospective or RCT studies are still needed to confirm our findings. Furthermore, the sample size was not estimated in this study because no previous study reported the fixation failure rate of the off-axis screw technique. In addition, the CT scans in the FEA were not calibrated by an individual phantom but were captured after the daily calibration of the department. However, the elasticity-density relation accuracy is satisfactory according to our previous validation test using cadaver bone [11].

## Conclusions

In the treatment of VFNFs, the complication rate was lowest for fixations with an off-axis screw and highest for parallel-screw fixation, and the complication rate for DHS fixation was intermediate between the other two. DHS had the greatest construct stability, producing significantly greater stiffness. On the other hand, the off-axis screw technique had the best interfragmentary stability, producing the lowest IFM and achieving the lowest fixation-failure rate. Assessment of the biomechanical characteristics of different fixation devices indicates that no inherent consistency exists between construct stability and interfragmentary stability. For VFNFs, analyzing interfragmentary stability in biomechanical experiments is more consistent with clinical prognosis than construct stability.

## Supplementary Information

**Additional file 1.** Table 1. Baseline information of the volunteers included in Subject-Specific FEA.

## Data Availability

The data and materials analyzed during the current study are available from the corresponding author on reasonable request.

## Abbreviations

VFNFs: Vertical femoral neck fractures
DHS: Dynamic hip screws
AVN: Avascular necrosis
NU: Non-union
FEA: Finite element analysis
IFM: Interfragmentary motion
