# Supplementary material for: Enhanced interfragmentary stability and improved clinical prognosis with use of the off-axis screw technique to treat vertical femoral neck fractures in nongeriatric patients
Source: J Orthop Surg Res. 2021 Jul 31;16:473. doi: 10.1186/s13018-021-02619-8 (PMC8325251; doi:10.1186/s13018-021-02619-8)
Supplement: Supplementary file 1 — Additional file 1. Table 1. Baseline information of the volunteers included in Subject-Specific FEA. [file 13018_2021_2619_MOESM1_ESM.docx]

| Patient | Age | Gender | Height  (m) | Weight  (Kg) | BMI (Kg/mm 2) | HU^1^ |  |
| --- | --- | --- | --- | --- | --- | --- | --- |
|  |  |  |  |  |  |  |  |
| 1 | 29 | F | 1.65 | 50 | 18.37 | 491.66 |  |
| 2 | 30 | F | 1.55 | 80 | 33.29 | 410.50 |  |
| 3 | 55 | F | 1.58 | 55 | 22.03 | 435.58 |  |
| 4 | 21 | M | 1.70 | 70 | 24.22 | 372.94 |  |
| 5 | 48 | M | 1.73 | 85 | 28.4 | 563.72 |  |
| 6 | 54 | M | 1.70 | 70 | 24.22 | 409.66 |  |
| 7 | 30 | M | 1.82 | 100 | 30.19 | 533.36 |  |
| 8 | 35 | M | 1.78 | 75 | 23.67 | 581.38 |  |

Table 1. Baseline information of the volunteers included in Subject-Specific FEA

1. Hu indicates the mean Hu value calculated with the method described in previous article (David, Journal Orthopedic Trauma 2018). Accordingly, a Hu value above 262 can be used to confirm the absence of osteoporosis.
